# Prevalence, Incidence, and Reversal Pattern of Childhood Stunting From Birth to Age 2 Years in Ethiopia

**DOI:** 10.1001/jamanetworkopen.2023.52856

**Published:** 2024-01-24

**Authors:** Frederick G. B. Goddard, Bezawit Mesfin Hunegnaw, Jonathan Luu, Sebastien J. P. A. Haneuse, Mesfin Zeleke, Yahya Mohammed, Chalachew Bekele, Daniel Tadesse, Meles Solomon, Delayehu Bekele, Grace J. Chan

**Affiliations:** 1 Department of Epidemiology, Harvard T. H. Chan School of Public Health, Boston, Massachusetts; 2 Department of Pediatrics and Child Health, St Paul’s Hospital Millennium Medical College, Addis Ababa, Ethiopia; 3 Department of Biostatistics, Harvard T. H. Chan School of Public Health, Boston, Massachusetts; 4 HaSET, St Paul’s Hospital Millennium Medical College, Addis Ababa, Ethiopia; 5 Maternal, Child and Adolescent health lead executive office, Federal Ministry of Health, Ethiopia; 6 Department of Obstetrics and Gynecology, St Paul’s Hospital Millennium Medical College, Addis Ababa, Ethiopia; 7 Department of Pediatrics, Boston Children’s Hospital, Harvard Medical School, Boston, Massachusetts

## Abstract

**Importance:**

Although there has been a reduction in stunting (low-height-for-age and low-length-for-age), a proxy of malnutrition, the prevalence of malnutrition in Ethiopia is still high. Child growth patterns and estimates of stunting are needed to increase awareness and resources to improve the potential for recovery.

**Objective:**

To estimate the prevalence, incidence, and reversal of stunting among children aged 0 to 24 months.

**Design, Setting, and Participants:**

This population-based cohort study of the Birhan Maternal and Child Health cohort in North Shewa Zone, Amhara, Ethiopia, was conducted between December 2018 and November 2020. Eligible participants included children aged 0 to 24 months who were enrolled during the study period and had their length measured at least once. Data analysis occurred from Month Year to Month Year.

**Main Outcomes and Measures:**

The primary outcome of this study was stunting, defined as length-for-age *z *score (LAZ) at least 2 SDs below the mean. *Z* scores were also used to determine the prevalence, incidence, and reversal of stunting at each key time point. Growth velocity was determined in centimeters per month between key time points and compared with global World Health Organization (WHO) standards for the same time periods. Heterogeneity was addressed by excluding outliers in sensitivity analyses using modeled growth trajectories for each child.

**Results:**

A total of 4354 children were enrolled, out of which 3674 (84.4%; 1786 [48.7%] female) had their length measured at least once and were included in this study. The median population-level length was consistently below WHO growth standards from birth to 2 years of age. The observed prevalence of stunting was highest by 2 years of age at 57.4% (95% CI, 54.8%-9 60.0%). Incidence of stunting increased over time and reached 51.0% (95% CI, 45.3%-56.6%) between ages 12 and 24 months. Reversal was 63.5% (95% CI, 54.8%-71.4%) by age 6 months and 45.2% (95% CI, 36.0%-54.8%) by age 2 years. Growth velocity point estimate differences were slowest compared with WHO standards during the neonatal period (−1.4 cm/month for girls and −1.6 cm/month for boys). There was substantial heterogeneity in anthropometric measurements.

**Conclusions and Relevance:**

The evidence from this cohort study highlights a chronically malnourished population with much of the burden associated with growth faltering during the neonatal periods as well as after 6 months of age. To end all forms of malnutrition, growth faltering in populations such as that in young children in Amhara, Ethiopia, needs to be addressed.

## Key Points

**Question:**

What is the burden, incidence, and reversal pattern of childhood stunting in Ethiopia?

**Findings:**

In this cohort study that included 3674 children, the prevalence of stunting at 2 years of age was 57.4%. Incidence of stunting was highest between the ages of 12 to 24 months (51.0%) and rates of stunting reversal were highest by 6 months of age (63.5%).

**Meaning:**

These findings suggest that understanding patterns of linear growth and onset of growth faltering or recovery serves as a critical input to meet national and global targets of ending malnutrition.

## Teaser

This cohort study analyzes the prevalence, incidence, and reversal of stunting among children aged 0 to 24 months in Ethiopia.

## Introduction

One central theme of the United Nations Sustainable Development Goals (SDGs) is child development, including Goals 2 and 4, which aim to end hunger and improve education.^1^ Nutritional status plays a major role in reaching these goals, because it is associated with both physical and cognitive development.^2^ Malnutrition continues to be a major public health problem and is the most important underlying factor associated with increased risk of illness and death, particularly in low-income countries and among young children.^3^ An estimated 53% of all deaths in children younger than 5 years are associated with malnutrition.^4^ Target 2.2. under Goal 2 of the SDGs set out to end all forms of malnutrition by 2030, including meeting targets on stunting in children younger than 5 years by 2025.^5^ Stunting is defined as low-length-for-age and low-height-for-age, and indicates chronic undernutrition.^6^ Stunting is considered to be an important indicator for both child physical and cognitive development,^7^ and is associated with environmental factors, such as living conditions and nutrition.^8^ In 2019, an estimated 144 million children younger than 5 years (21%) globally were stunted.^9^

To meet the SDGs and end all forms of malnutrition, a wealth of evidence has been generated to understand how and when to intervene. A recent collection of research by the Ki Child Growth Consortium^10,11^ that combined anthropometric data from over 100 000 children enrolled in 35 longitudinal cohorts across 15 low- and middle-income countries found that the incidence of stunting peaks early between birth and 3 months of age. Recovery from stunting was rare, with only 5% of children reversing their stunting status any given month.^11^ The prevalence of child stunting in sub-Saharan Africa remains high, including in Ethiopia.^12^ Although there has been a reduction in stunting over the last 2 decades, in 2016, an estimated 38% of children younger than 5 years in Ethiopia were stunted.^13^ There is high spatial heterogeneity of stunting within Ethiopia,^14^ with the highest prevalence of stunting occurring in the Amhara region.^15^ As part of the World Health Assembly global nutrition targets, Ethiopia aims to reduce stunting in children younger than 5 years to 27% by 2025 with an expected mean annual reduction rate of 6%.^16,17^ Current estimates show that the country is off track following a mean annual reduction rate of only 2.4%.^18^

To achieve these goals, rigorous studies that not only describe the magnitude of the problem but also the dynamics of growth and changes through childhood are imperative. Accurate depiction of the growth process that includes analysis of growth trajectories and patterns among populations and subgroups is critical to understanding and identifying key time points that predispose children to growth faltering. There are limited longitudinal data on stunting, particularly from underserved populations in low resource settings. Although the recent studies by the Ki Child Growth Consortium included large, pooled data sets of longitudinal anthropometric data, they did not include data from Ethiopia. Infants younger than 6 months of age are often excluded from nutrition surveys and marginalized in nutrition programs,^19^ even though this time period is characterized not only by maximal growth velocity but also by vulnerability to nutrition related events and insults. The Birhan maternal and child health (MCH) cohort in Amhara, Ethiopia, can fill some of these gaps, with longitudinal anthropometry data from birth until the child reaches their second birthday.^20^

## Methods

### Study Design

This cohort study was approved by the institutional review boards of St Paul’s Hospital, Ethiopia, and Boston Children’s Hospital and followed the Strengthening the Reporting of Observational Studies in Epidemiology (STROBE) reporting guideline. Written informed consent was obtained from mothers prior to enrollment. This study was nested within the Birhan field site, a platform for community and facility-based research and training that was established in 2018, with a focus on maternal and child health.^21^ Nested in the site is an open cohort, the Birhan MCH cohort, which enrolls approximately 2000 pregnant women and their newborns per year with longitudinal follow-up over the first 2 years of life and household data linked with health facility information.^20^

### Study Population

All pregnant women and mothers (guardians) of children younger than 2 years of age residing in the study area who provided consent were enrolled to the parent cohort. From December 2018 to November 2020, children were enrolled through both a birth cohort (ie, enrolled at birth) and a nonbirth cohort (ie, enrolled after birth). Children enrolled to the study were followed until they reached 2 years of age.

### Data Collection

A custom length board, standardized against the infant and child ShorrBoard (Weigh and Measure) and Seca 417 infantometer (Seca) were used for length measurements. Length measurements were performed by study nurses and midwives who were given lessons on theory and participated in lab sessions on WHO standard child growth assessment procedures to measure length. Measurements were recorded to the nearest 0.1 cm. Length was measured as single measurements (rather than averaging repeated measures for patient acceptability) during scheduled child follow-up visits at birth, on days 6, 28, and 42 after birth; and at 6 months, 12 months, and 24 months of age; as well as from health facility visits (ie, outpatient visits or hospital admissions).

### Outcomes

The primary outcome of the study was stunting, defined as length-for-age *z* score (LAZ) less than 2 SDs below the mean. We generated LAZ with length, age, and sex data using WHO growth standards.^22^ *Z *scores were used to determine the prevalence, incidence, and reversal of stunting at each key time point. International Fetal and Newborn Growth Consortium for the 21st Century standards^23,24^ were used to generate LAZ scores at birth and during the neonatal period; the standards were generated using data from low-income and middle-income countries, and adjust for gestational age at birth, in contrast with WHO standards^22^ that assume all babies are born at 40 weeks gestation and, hence, can overestimate stunting prevalence at birth and during the neonatal period. We excluded anthropometric measurements if (1) the *Z* scores fell outside the biologically plausible range defined by the WHO (LAZ <−6 or LAZ >6) or (2) the measurements were abstracted from facility charts after birth because these measurements were not validated by our data collectors. We compared the median (IQR) length of children enrolled in the Birhan cohort to WHO global growth standards^22^ at key time points. Growth velocity was determined in centimeters per month between key time points and compared with global WHO standards for the same time periods.^25^ We defined reversal of stunting as an LAZ no longer less than 2SDs below the mean. Definitions of low birth weight, preterm birth, and estimation of gestational age can be found in the eAppendix in Supplement 1.

Key time points were defined as birth, 4 weeks, 6 weeks, 6 months, 12 months, and 24 months. Birth length measurements were defined as those that were collected within the first week of life. For measurements at the end of the neonatal period and at 6 weeks, we included those taken at 4 weeks and 6 weeks (±1 week) after birth. For the other key time points (6 months, 12 months, and 24 months) we included measurements taken within 4 weeks of each time point.

### Statistical Analysis

Empirical length measurements were stratified by sex to describe population median (IQR) length at birth, 4 weeks (ie, the end of the neonatal period), 6 weeks, 6 months, 12 months, and 24 months of age. Differences in medians between key time points were used to calculate monthly growth velocity. Medians at each key time point and velocities between key time points were then compared with WHO standards. For incidence calculations, the sample population for each time period (defined as 2 adjoining key time points) was defined as all children that were at risk of stunting (ie, not already having stunted growth by the beginning of that time period) and have available anthropometric measurements between 2 adjoining key time points. Similarly, for reversal, the sample population for each time period was defined as all children that had the chance of reversing their stunting (ie, already having stunted growth by the beginning of that time period) and have available anthropometric measurements at the beginning and end of the time period. To expand on our incidence and reversal analysis, we calculated incidence and reversal both sequentially (ie, comparing birth with 4 weeks and comparing 4 weeks with 6 weeks) and using birth as a baseline (ie, comparing birth to 4 weeks and birth to 6 weeks).

To consider potential measurement error for length in our findings, we used a mixed effects model to determine potential outliers that should be removed from the analysis. Using LASSO regression to determine significant covariates to include in the models, we considered 6 different models that used a mix of fixed and random age variables and intercepts, quadratic splines, and piecewise-linear splines. Our final model included a piecewise-linear spline of age for the fixed effect, and an intercept and slope of standardized age for the random effects. We determined this model was the best fit after comparing Akaike information criterion, Bayes information criterion, and deviance diagnostic criteria. Using the predicted outcomes and estimated standard deviation from the model, we removed observed points that were not within 1.5 SDs of fitted trajectories. After these measurements were removed from the data set, prevalence, incidence, and reversal estimates were recalculated. Confidence intervals for all proportion estimates were calculated using the Agresti-Coull method. All analyses were completed in R Version 4.2.3 (R Project for Statistical Computing).^26^ Data analysis occurred from July 2021 to July 2022.

## Results

**Figure 1.**
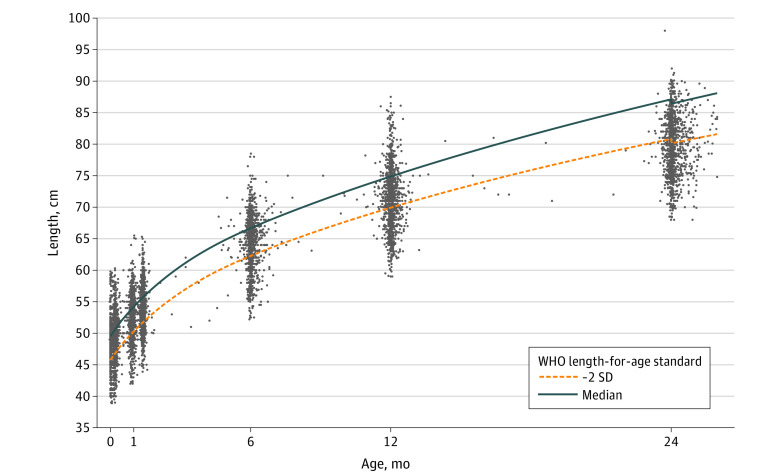
Distribution of Length Measurements Compared With World Health Organization (WHO) Standards The figure shows the distribution of length measurements for boys and girls combined.

We enrolled 4354 children younger than 2 years of age into the MCH cohort study, among which 3674 (84.4%; 1786 [48.7%] female) had their length measured at least once and were included in this study (Table 1). Among children included, 3037 (82.7%) resided in rural areas and. Among 2350 children with gestational age data, 360 (15.3%) were born preterm, and among 1732 children with birth weight data, 156 (9.0%) had low birth weight. Over the course of the study, length measurements were taken a mean (range) of 2.6 (1-8) times for each child. Length measurements were clustered around the scheduled follow-up visits (Figure 1). A total of 206 children whose measurements were outside ±6 LAZ were excluded.

**Table 1.** Population Summary Characteristics

Characteristic	Participants, No./Total No. (%)
Birth cohort	2592/4354 (59.5)
Nonbirth cohort	1762/4354 (40.5)
Children with length data available	3674/4354 (84.4)
Residing in rural areas	3037/3674 (82.7)
Sex	
Female	1786/3670 (48.7)
Male	1884/3670 (51.3)
Preterm births	360/2350 (15.3)
Low birth weight	156/1732 (9.0)
Singleton births	3557/3674 (96.8)
Length measurements, mean (SD) [range] (n = 3674)	2.6 (1.6) [1-8]

Median (IQR) length was lower in the study population compared with global WHO standards at all key time points defined for this study (ie, birth, the end of the neonatal period [4 weeks], 6 weeks, 6 months, 12 months, and 24 months) (Table 2). The point estimates of the differences were smallest at birth (−0.1 cm for girls and −0.9 cm for boys); thereafter, there was slower monthly growth velocity point estimate differences compared with WHO standards that were most pronounced during the neonatal period (−1.4 cm/month for girls and −1.6 cm/month for boys). After the neonatal period, the point estimate difference in growth velocity was smaller for girls between 4 weeks and 6 weeks (−0.7 cm/month) and between 6 weeks and 6 months (−0.2 cm/month). There was no difference in growth velocity point estimates for boys in the MCH cohort compared with WHO standards between the end of the neonatal period and 6 weeks. Differences in growth velocity were between −0.2 cm/month and −0.3 cm/month thereafter, resulting in the largest difference in point estimates between median length in the MCH cohort study compared with WHO standards by 2 years of age (−6.6 cm for girls and −7.8 cm for boys).

**Table 2.** Length-for-Age and Length at Key Times Points for Girls and Boys Compared With WHO Global Standards

Measurement	Girls						Boys					
	Birth (n = 882)	4 wk (n = 787)	6wk (n = 787)	6 mo (n = 825)	12 mo (n = 863)	24 mo (n = 688)	Birth (n = 938)	4 wk (n = 775)	6 wk (n = 778)	6 mo (n = 830)	12 mo (n = 906)	24 mo (n = 733)
Length-for-age												
Birhan *z* score, median (IQR)	0.00 (−1.04 to 1.17	−0.64 (−1.74 to 0.70)	−0.55 (−1.60 to 0.71)	−0.62 (−1.79 to 0.40)	−1.17 (−2.42 to −0.21)	−2.11 (−3.17 to −1.10)	−0.19 (−1.22 to 0.80	−0.79 (−2.05 to 0.43)	−1.07 (−2.15 to 0.34)	−1.21 (−2.20 to −0.15)	−1.63 (−2.85 to −0.72)	−2.54 (−3.70 to −1.57)
Birhan median (IQR) cm	49.0 (47.3 to 50.2)	52.0 (50.0 to 54.0)	53.4 (51.5 to 55.6)	64.0 (61.5 to 66.2)	71.0 (68.0 to 73.5)	79.8 (76.4 to 83.0)	49.0 (47.5 to 50.5)	52.0 (50.0 to 54.3)	53.8 (52.0 to 56.0)	65.0 (62.5 to 67.0)	72.0 (69.0 to 74.0)	80.0 (76.6 to 83.0)
WHO standard, cm	49.1	53.4	55.1	65.7	74.0	86.4	49.9	54.4	56.2	67.6	75.7	87.8
Birhan vs WHO difference, cm	−0.1	−1.4	−1.7	−1.7	−3.0	−6.6	−0.9	−2.4	−2.4	−2.6	−3.7	−7.8
Growth velocity, cm/mo												
Birhan	NA	3.3	3.0	2.3	1.2	0.7	NA	3.3	3.9	2.4	1.2	0.7
WHO standard	NA	4.7	3.7	2.5	1.4	1.0	NA	4.9	3.9	2.6	1.4	1.0
Birhan vs WHO difference	NA	−1.4	−0.7	−0.2	−0.2	−0.3	NA	−1.6	0	−0.2	−0.2	−0.3

Abbreviations: NA, not applicable; WHO, World Health Organization.

We investigated the potential for measurement error in our data and found variation in longitudinal length measurement for some individuals. To determine which points to exclude in a repeated prevalence, incidence, and reversal analysis, we compared the fitted trajectories for each individual with observed data points. Observed lengths that were 1.5 SDs away from fitted growth trajectories for 922 measurements were removed from the modeled prevalence, incidence, and reversal analysis (eTable in Supplement 1).

**Figure 2.**
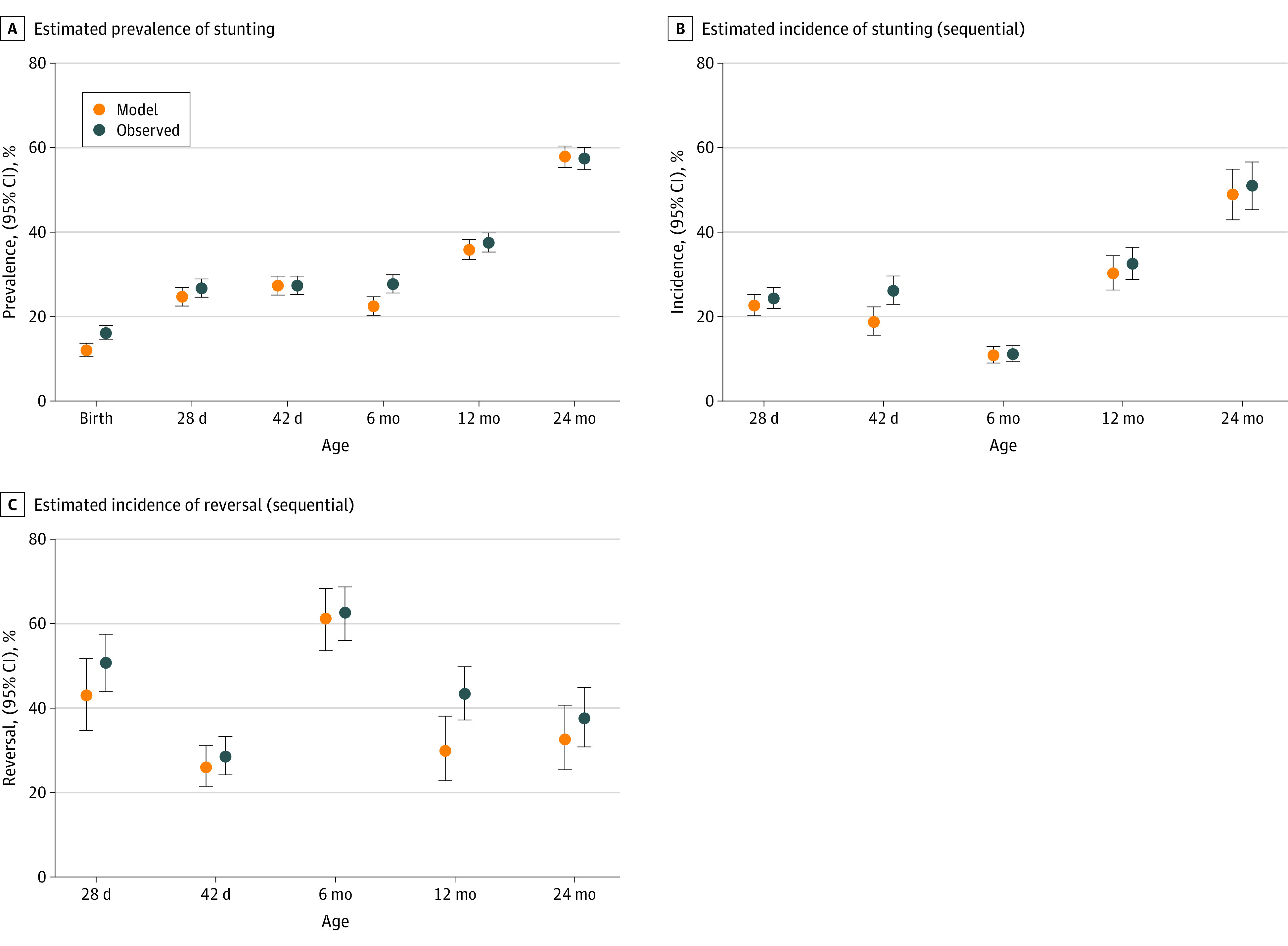
Prevalence, Incidence, and Reversal of Stunting

Using observed data, the prevalence of stunting at birth was 16.1% (95% CI, 14.5%-17.9%) and showed a gradual increase with a sharp rise from the age of 6 months to 24 months, reaching an observed prevalence of 57.4% (95% CI, 54.8%-60.0%) at 24 months. Observed prevalences from the Birhan data were higher than modeled values at birth and at 6 months, whereas prevalences were similar at other key time points (Figure 2A).

The incidence, indicating that children were newly stunted at a given key time point compared to the previous time point, followed a similar pattern to the prevalence except for the 6-week time point (Figure 2B). Among 1149 newborns who were not stunted at birth, 279 (24.3%) experienced observed incident stunting within the first month of life. The highest incidence of cases was observed between the ages of 12 to 24 months (151 out of 296 individuals [51.0%]; 95% CI, 45.3%-56.6%) followed by the 6 to 12 months period (192 of 591 individuals [32.5%]; 95% CI, 28.8%-36.4%). Observed incidence estimates were, for the most part, higher than modeled. Further analysis using birth as a common baseline time point showed a steady increase in incidence up to 2 years of age (eFigure 1 in Supplement 1). Overall, among 531 children who were not stunted at birth, 205 (38.6%) had incident stunting at 1 year of age and 331 of 585 children with data (56.6%) had new onset stunting by 2 years of age.

The marked difference in incidence between the 2 time points compared with difference in prevalence among these same time points indicates the presence of a dynamic incidence and reversal process through time. Rates of reversal (both sequentially when compared to a prior time point and using birth as baseline) were highest by 6 months of age (Figure 2C and eFigure 2 in Supplement 1). Among children who were already stunted at birth and thus had a chance to reverse stunting later in life, 80 of 126 children with data (63.5%; 95% CI, 54.8%-71.4%) were able to do so by 6 months of life and 59 of 96 children with data (61.5%; 95% CI, 51.5%-70.6%) reversed stunting by the end of the first year of life (eFigure 2 in Supplement 1). By the age of 2 years, 47 of 104 children with data (45.2%; 95% CI, 36.0%-54.8%) who were stunted at birth no longer had stunted growth.

## Discussion

Our findings in this cohort study provide evidence that median population-level length among children in our study sample are consistently below global standards from birth to 2 years of age. Monthly growth velocity was slowest compared with global standards during the neonatal period before stabilizing until the child reached 6 months of age, and then slowing again thereafter. Incidence of stunting followed a similar trajectory, suggesting an increasing incidence of children with newly stunted growth over time. Reversal of stunting was a frequent observation with the highest rate of reversal occurring between birth and 6 months of age.

These findings suggest that among this study population, most loss in potential linear growth occurred during the neonatal period, and then again, after 6 months of age. An elevated burden in malnutrition after 6 months was also documented in the 2016 Ethiopia Demographic and Health survey (EDHS),^13^ and may be associated with household food insecurity, poor infant and young child feeding practices, lack of food diversity and frequency, as well as an increased exposure to enteric pathogens and infectious diseases.^27,28,29^ The prevalence of stunting at 2 years of age in our study was higher than the 2016 EDHS, which found a 47.2% prevalence of stunting among children aged 0 to 59 months in the same region.^15^

Our stunting prevalence findings are consistent with those from the Ki Child Growth Consortium, which combined longitudinal anthropometric data from multiple cohorts, (but did not include data from Ethiopia) where a steady increase was observed in the prevalence of stunting until 2 years of age.^11^ However, in contrast with findings from the Ki Child Growth Consortium, we found that incidence of stunting in this population did not peak during the first 3 months of life, but rather continued to increase throughout the first 2 years of life. Stunting reversal was much higher in our study population, particularly early in life from birth to 6 months and 12 months of age. This finding may be reflective of the impact of various initiatives in place throughout the country to reduce stunting, which include nutritional interventions (particularly predominant breastfeeding in the first 6 months of life), improved health care access, sanitation, and education.^30^ Additionally, the infancy period is also marked by the greatest potential increment in linear growth, thereby presenting higher likelihood of favorable interventions associated with reversal changes. However, the high prevalence and incidence of stunting suggests that even when stunting status is reversed, a substantial number of children relapse.

This study also found evidence of within-child heterogeneity in longitudinal length measurements that may be due to measurement error. Challenges associated with measuring length among newborns, infants, and toddlers have been well documented.^31,32^ In this study, we excluded measurements that were not biologically plausible as defined by WHO standards. We also developed modeled trajectories with previously validated methodologies using linear spline multilevel models that have knots at key time points,^33,34,35^ to validate the observed prevalence, incidence and reversal findings. Excluding outliers did not substantially alter prevalence, incidence, or reversal findings. Observed values were slightly higher than modeled estimates, especially in the earlier ages, implying that our observations may have overestimated the outcome, which may possibly be associated with difficulty in measuring length in the younger ages.

### Limitations

The findings of this study should be interpreted with its limitations in mind. We saw heterogeneity of length measurements for individual children, both at the same time point and across 2 time points. We were also limited in the number of measurements we had per child and each child contributed a different number of observations to this analysis due to late enrollments in the non-birth cohort, intermittently missed scheduled visits, differences in the prevalence of unscheduled health care-seeking visits, or missing data from the pausing of data collection due to COVID-19. We also recognize that incidence and reversal events or relapses may occur within a single, longer study time frame, which may have been identified with shorter time windows especially in the post infancy period. However, this study provides several important contributions to the literature. It outlines longitudinal evidence on the burden, onset and reversal of growth faltering in Amhara, Ethiopia, the region identified by the 2016 EDHS as having the highest burden of malnutrition in the country.^13^ This study also provides evidence of linear growth at birth and during the neonatal period, where available data are more limited.

## Conclusion

There is growing recognition that growth faltering is a dynamic process with the burdens of undernutrition not limited to those that pass the threshold for stunting at a defined point in time.^6^ Instead, it is worth noting, that the evidence from this study suggests that this population is chronically malnourished compared with global standards. To meet the United Nations SDGs and end all forms of malnutrition, growth faltering in populations such as that in children younger than 2 years in the North Shewa Zone of Amhara, Ethiopia, needs to be addressed.

## Notes

Supplement 1. **eAppendix. **Definitions of Low Birth Weight (LBW), Preterm Birth and Estimation of Gestational Age
eReferences
**eTable 1.** Number of Observations **eFigure 1.** Estimated Incidence of Stunting (Common Baseline–Birth) **eFigure 2.** Estimated Incidence of Reversal (Common Baseline–Birth)

Supplement 2.
Data Sharing Statement
