# Supplementary material for: Prevalence, Incidence, and Reversal Pattern of Childhood Stunting From Birth to Age 2 Years in Ethiopia
Source: JAMA Netw Open. 2024 Jan 24;7(1):e2352856. doi: 10.1001/jamanetworkopen.2023.52856 (PMC10809014; doi:10.1001/jamanetworkopen.2023.52856)
Supplement: Supplement 1. — eAppendix. Definitions of Low Birth Weight (LBW), Preterm Birth and Estimation of Gestational Age eReferences eTable 1. Number of Observations eFigure 1. Estimated Incidence of Stunting (Common Baseline–Birth) eFigure 2. Estimated Incidence of Reversal (Common Baseline–Birth) [file jamanetwopen-e2352856-s001.pdf]

Supplemental Online Content

Goddard FB, Hunegnaw BM, Luu J, et al. Prevalence, incidence, and reversal pattern of childhood stunting from birth to age 2 years in Ethiopia. *JAMA Netw Open.* 2024;7(1):e2352856. doi:10.1001/jamanetworkopen.2023.52856

eAppendix. Definitions of Low Birth Weight (LBW), Preterm Birth and Estimation of Gestational Age
eReferences
eTable. Number of Observations
eFigure 1. Estimated Incidence of Stunting (Common Baseline–Birth)
eFigure 2. Estimated Incidence of Reversal (Common Baseline–Birth)

This supplemental material has been provided by the authors to give readers additional information about their work.

© 2023 Goddard FBG et al. *JAMA Network Open.*

eAppendix. Definitions of Low Birth Weight (LBW), Preterm Birth and Estimation of Gestational Age

We described population characteristics for children included in the study. Low birth weight (LBW) was defined as live births born less than 2,500 grams. Preterm birth was defined as live births before 37 weeks of gestation or fewer than 259 days from the first date of a woman's last normal menstrual period LMP. Gestational age at birth was estimated using a gestational age hierarchy, adapted from the American College of Obstetricians and Gynecologists guidelines[1] and Global Library of Women's Medicine[2] based on the best available method from ultrasound measurements, maternal report of LMP, maternal report in months, and fundal height.

eReferences

1. Methods for estimating the due date. Committee Opinion No. 700. American College of Obstetricians and Gynecologists. Obstet Gynecol. 129(e150-4):e150-4.
2. MacGregor S, Sabbagha, R. Assessment of Gestational Age by Ultrasound. Glob libr women's med. 2008.

eTable. Number of Observations

	Birth	Day 28	Day 42	6 months	12 months	24 months
Number of observations	2979	1565	1569	1669	1780	1422
Number of removed points	239	87	91	257	227	21
Number of observations after removal	2740	1478	1478	1412	1553	1401

eFigure 1. Estimated Incidence of Stunting (Common Baseline–Birth)

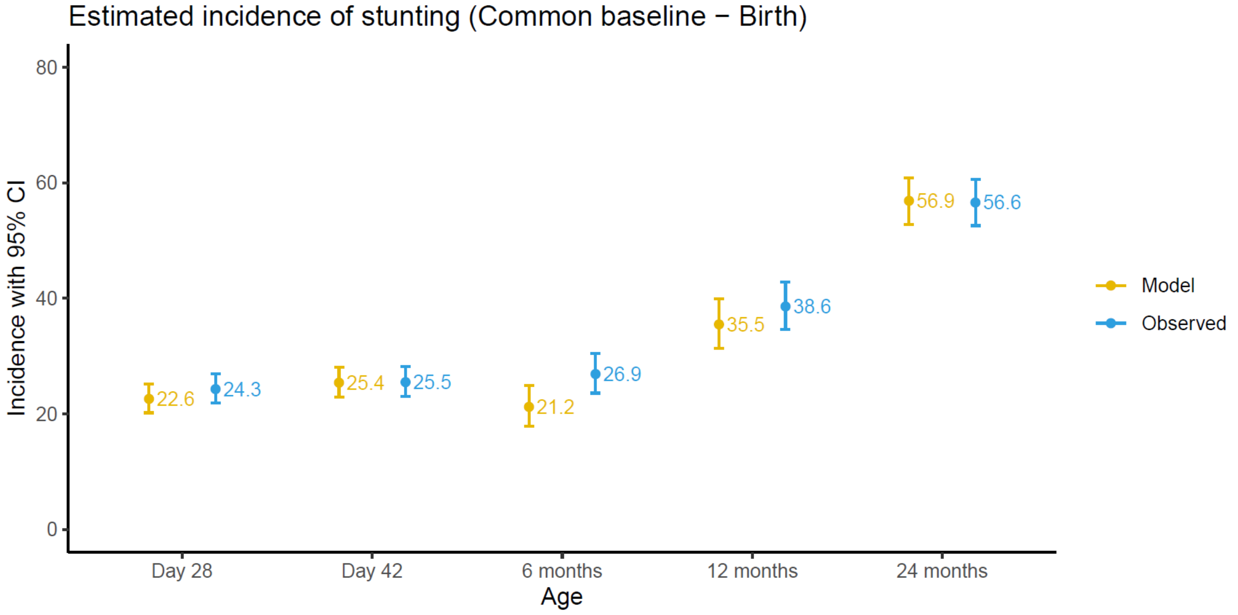

eFigure 2. Estimated Incidence of Reversal (Common Baseline–Birth)

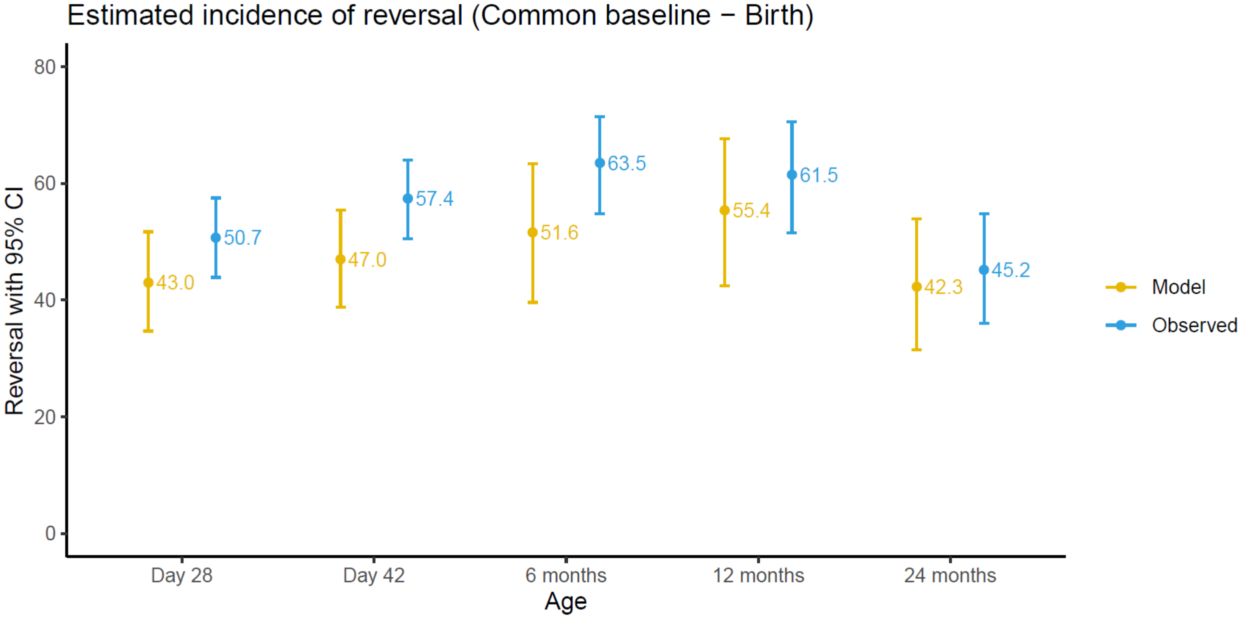

© 2023 Goddard FBG et al. *JAMA Network Open.*
