# Supplementary material for: Prevalence, Incidence, and Reversal Pattern of Childhood Stunting From Birth to Age 2 Years in Ethiopia
Source: JAMA Netw Open. 2024 Jan 24;7(1):e2352856. doi: 10.1001/jamanetworkopen.2023.52856 (PMC10809014; doi:10.1001/jamanetworkopen.2023.52856)
Supplement: Supplement 2. — Data Sharing Statement [file jamanetwopen-e2352856-s002.pdf]

Data Sharing Statement

Goddard. Prevalence, Incidence and Reversal Pattern of Childhood Stunting From Birth to Age 2 Years in Ethiopia. *JAMA Netw Open*. Published January 24, 2024.
doi:10.1001/jamanetworkopen.2023.52856

Data
Data available: Yes
Data types: Deidentified participant data
How to access data: Data use is governed by the Birhan Data Access Committee (DAC) and follows Birhan's data sharing policy. All researchers who wish to access Birhan data can complete a Birhan data request form and submit it for decision by the Birhan DAC. Datasets will only be provided with de-identified data to maintain confidentiality of study participants. Dataset will be available in dataverse repository in October 2023 and URL will be made available at that time.
When available: With publication

Supporting Documents
Document types: Statistical/analytic code
How to access documents: Analytic code will be made available on Birhan github
https://github.com/birhan-data/data-public
When available: With publication

Additional Information
Who can access the data: Data will be made available to researchers whose proposed use of the data has been approved by the Birhan Data Access Committee.
Types of analyses: Data will be made available for purposes approved by the Birhan Data Access Committee.
Mechanisms of data availability: Data will be made available after approval of proposal and data request submitted to Birhan Data Access Committee and with a signed data access agreement.
